# Supplementary material for: Antarctic ozone hole modifies iodine geochemistry on the Antarctic Plateau
Source: Nat Commun. 2021 Oct 5;12:5836. doi: 10.1038/s41467-021-26109-x (PMC8492625; doi:10.1038/s41467-021-26109-x)
Supplement: Supplementary file 1 — Supplementary material [file 41467_2021_26109_MOESM1_ESM.pdf]

SUPPLEMENTARY INFORMATION

Antarctic ozone hole modifies iodine geochemistry on the Antarctic Plateau

[1,2,*]Andrea Spolaor, [2,3]Francois Burgay, [4]Rafael P. Fernandez, [1,2]Clara Turetta, [5]Carlos A. Cuevas, [6]Kitae Kim, [7]Douglas E. Kinnison, [7]Jean-François Lamarque, [1,2]Fabrizio De Blasi, [1,2]Elena Barbaro, [5,8]Juan Pablo Corella, [9,10]Paul Vallelonga, [11]Massimo Frezzotti, [1,2]Carlo Barbante and [5*]Alfonso Saiz-Lopez

[1]*Institute of Polar Sciences, ISP-CNR, Campus Scientifico Via Torino 155, 30172 Mestre, Venice, Italy.*

[2]*Department of Environmental Sciences, Informatics and Statistics, University Ca'Foscari of Venice, via Torino, 155 - 30172 Venice-Mestre, Italy*

[3]*Paul Scherrer Institute, Laboratory of Environmental Chemistry, 5232 Villigen PSI, Switzerland*

[4]*Institute for Interdisciplinary Science, National Research Council (ICB-CONICET), FCEN-UNCuyo, Mendoza, 5501, Argentina*

[5]*Department of Atmospheric Chemistry and Climate, Institute of Physical Chemistry Rocasolano, CSIC, Madrid, Spain*

[6] *Korea Polar Research Institute, Incheon 21990, Korea*

[7] *National Center for Atmospheric Research, Boulder, CO, USA*

[8]*CIEMAT, Department of the Environment (DMA), Madrid, Spain*

[9]*Physics of Ice, Climate and Earth, Niels Bohr Institute, University of Copenhagen, Tagensvej 16, Copenhagen N2200, Denmark*

[10]*UWA Oceans Institute, University of Western Australia, Crawley WA 6009, Australia*

[11]*Department of Science, University of Roma Tre, Largo S. Leonardo Murialdo, 1 00146 Roma, Italy.*

S1 - Shallow Ice core and snow pit chronology

Defining a precise ice core chronology in the Antarctic plateau is complex mainly due to the very low snow accumulation and the possibility that some annual layers are removed by wind scouring events. The 13.72 m shallow ice core chronology is based on: a) the surface snow accumulation and b) the comparison with the ice age chronology based on the identification of past volcanic horizons calculated by Gautier et al. 2016 [1] from five ice cores collected in 2010 at Dome C. In details, the ice core depth used for this study was converted into meters of water equivalent using the available average density profile for Dome C[2] to fit the depth profile calculated by Gautier et al.. Then, to match our core collected in 2012 with the 5 ice core chronology calculated by Gautier et al. 2016, we used the annual snow accumulation data (averaged to 27 mm w.e. per year) to fill the two years gap between the studies. Afterwards the age chronology obtained from Gautier et al. has been applied to our ice core. The shallow ice core age scale calculated and proposed for this study, with the associated uncertainty, is reported in the Table S1.

We followed the same approach to date the snow pit samples collected in December 2013 (Sp2013 in the main text) and in December 2017 (Sp2017 in the main text) to match them with the shallow ice core.

S2 - Snow pits sampling and analysis.

The 1.3 m-deep snow pit (Sp2013) was dug 3 km far from the station and the samples were analysed for I and Na while the 4 m-deep snowpit (Sp2017) was dug 7 km from Concordia and the samples analysed for Na, I and major ions. Additionally, snow density, hardness index and snow grain size were also recorded from the Sp2017.

The snow pits were sampled with the precaution necessary for the trace elements analysis[3,4]. The snow pit was dug manually using aluminium shovels and before sampling, the snow pit wall was cleaned to remove possible contamination from the aluminium shovel with a dedicated pre-cleaned polyethylene scoop. The snow wall was sampled using polyethylene pre-cleaned 50 mL vials with a depth interval of 3 cm inserting the tube directly into the snow wall. Density profile was measured with 10 cm resolution while hardness and snow grain were measured from each identified snow layer. Iodine and sodium analyses in the snow pit were conducted on melted and not acidified samples with the same analytical procedure used for the ice core samples (described in method section).

Ion chromatograph (IC, Thermo Scientific Dionex™ ICS-5000, Waltham,MA, USA) coupled with a single quadrupole mass spectrometer (MS,MSQ Plus™, Thermo Scientific, Bremen,

Germany) was used to analyze major ions including acetate, methanesulphonic acid (MSA), chloride, ammonium and nitrate[5]. The identification was performed using an IC (ThermoScientific™ Dionex™ ICS-5000, Waltham, US) equipped with an anionic exchange column (Dionex Ion Pac AS 19 2 × 250 mm) and a guard column (Dionex Ion Pac AG19 2 × 50 mm). Sodium hydroxide (NaOH), used as mobile phase, was produced by an eluent generator (Dionex ICS 5000EG, Thermo Scientific). The NaOH gradient with a 0.25 mL min^{-1} flow rate was: 0-6 min at 15 mM; 6-15 min gradient from 15 to 45 mM; 15-23 min, column cleaning with 45 mM; 23–33 min; equilibration at 15 mM. The injection volume was 100 µL. A suppressor (ASRS 500, 2 mm, Thermo Scientific) removed NaOH before entering the MS source. The IC was coupled to a single quadrupole mass spectrometer (MSQ Plus™, Thermo Scientific™) with a negative electrospray source (ESI) that operated with a temperature of 550 °C and a needle voltage of 3 kV. The mass spectrometer parameters are reported in Barbaro et al. 2017[5].

S3 - Statistical analysis

S3.1 - Statistical tests for tipping point detection

We used the CPAt method to detect the tipping point[6]. CPAt is a detection method that uses an interaction of cumulative sum (CUSUM), mean square error (MSE) and bootstrapping to identify the change point using several iterations. CUSUM is calculated as:

$$S_i = S_{i-1} + (X_i - \bar{X})$$

where S_i is cumulative sum value at time i and $\bar{X}$ is the average value of the time series. The change in the CUSUM slope identifies the probable tipping point. The second estimator to identify the changes is MSE that is calculated as follow:

$$MSE = \sum_{i=1}^{m}(X_i - \bar{X}_a)^2 + \sum_{i=m+1}^{n}(X_i - \bar{X}_b)^2$$

where $\bar{X}_a$ and $\bar{X}_b$ are respectively the average values of the period after and before the change point. The best estimator of the change point is the value of m that minimizes the MSE. To determine a confidence level about the change point, a bootstrap analysis can be performed using an estimator of the magnitude of the change (M). The analysis is based on the idea of bootstrap samples. They represent a large number of new datasets with the same original data but with different random order for each one. If there are no

changes into the series, any type of reordering has most likely the same behaviour of the CUSUM or MSE. Using a large number of bootstrap samples (N), a confidence level (CL) for each change point can be calculated comparing the number of bootstraps (P) for which M_{diff}^{0} is less than M_{diff}:

$$CL\,\% = 100\,\frac{M}{P}$$

Additional advantages of CPAt are the identification of time range around the tipping point and the chance to identify multiple change points in data series. With CPAt tool we can choose the confidence interval for the time of change to identify how well the time of the change has been identified. The higher the confidence interval (eg 99%), the most likely larger the time interval around the tipping point. About the identification of multiple change points, once a first change is detected with its confidence level, the dataset is split into two different subsets and for each one CUSUM, MSE and bootstrapping analysis is performed again. The iterative analysis is complete when no more significant change points are detected. In the end, the set of detected change points is again estimated and a backward elimination procedure is performed in order to eliminate the change points of no significance. We have used the Change-Point Analyzer tool [6] to perform the calculations.

S3.2 - Correlation test

We have used the Pearson correlation (R) of the datasets before and after the identified change point as an additional confirmation of the occurrence of the tipping point in 1974. After splitting the identified dataset into two segments, for each one we have calculated R and the associated p-value by converting R-value to a t-statistic.

$$t = \frac{|R|\,\sqrt{n-2}}{\sqrt{1-R^2}}$$

S3.3 - Dome C iodine ice core concentration and stratospheric ozone.

We computed the correlation between annual ice core measured [I] with modelled sunlit total ozone columns above Dome C (TOC^{DC}). The correlation obtained for the ozone hole period 1975 – 2011 is: r = 0.398, p-value = 0.015 while for the pre-ozone hole period 1950 – 1974 the correlation obtained is r = − 0.1, p-value = 0.65.

S3.4 - Change in [I] ice core and AF^{300}.

The negative association between annual ice core [I] and AF^{300} is confirmed by the Pearson coefficient that becomes negative and more significant during the ozone hole period, while is not significant for the pre-ozone hole period (post-1975 r = -0.401, p-value = 0.015; pre-1975 r =-0.18, p-value = 0.93).

S3.5 - Comparison of I and Na concentration merged records (Na_M and I_M)

Thanks to the availability of the data from two snow trenches in an area close to the drilling site (see S2 for further details), we validated the ice core chemical analysis with these samples. Merging the dataset was necessary to complete the sodium record that presents a gap in the period 1989-1997 due to analytical failure. The good agreement among the three records allowed us to merge the three datasets for both Na and I. We used the same matching procedure for Na and I based on the chronology described in section S1. First, we calculated the annual average of each record for the time alignment among the three datasets. From the three annual time series, we extracted the spatial average annual value as the mean of the available data for each time step. In addition, we calculated the spatial variability for each year considering the deviations of each record from the average value. After obtaining the merged record that include the ice core and snow pit records, we calculated the correlation between merge Na (Na_M) and I (I_M) before and after 1975. A significant statistical correlation exists between I_M and Na_M during the pre-ozone hole period (1800 to 1975; r = 0.44; p-value< 0.0001) but not for the period 1975 to 2017 (r = -0.1; p-value = 0.54).

S4. Comparison with other iodine ice core records

Only two ice core iodine records exist in Antarctica: one from Talos Dome (72°49'S, 159°11'E, 2315 m a.s.l.) and the other from Law Dome (66.46ºS, 112.48ºE, 1395 m a.s.l.) (Figure S1). The first one has a low temporal resolution due to the relatively scarce annual accumulation (80 mm we yr^{-1}) since the aim was to investigate the multi-millennia variability of iodine species and only a few samples covered the Holocene[7]. On the contrary, the Law Dome ice core (740 mm we yr^{-1}) covered the period from 1929 to 1988 at a sub-annual resolution and does not present a decrease in iodine concentration from the late 70s to the early 80s[8]. We argue that this distinct behaviour is related to the mean location height, the environmental conditions and the transport from the coastal areas at Law Dome, which are quite different to the ones at Dome C. Furthermore, the higher snow accumulation and the continuous direct influences of coastal emissions prevailing at Law Dome, likely masked any increase in iodine photochemical re-emission from the snowpack. In addition, note that the Law Dome record ended in 1988, when the CFCs and halons influence on stratospheric ozone was not at its maximum [9] and the geographical extension of the ozone hole area was still confined within the inner Antarctic plateau (see section S6).

In the Arctic, the Greenland Renland ice core (71.30°N, 26.72°W, 2315 m a.s.l., snow accumulation about 450 mm we yr^{-1}) iodine record overlaps the entire period described in this work and

allows a comparison between the Northern and the Southern Hemispheres. However, the results obtained from the Renland ice core [10] are opposite to those retrieved from Dome C (Figure S1). This opposite hemispheric behaviour can be explained by three main facts: a) the much lower Arctic stratospheric ozone depletion due to the anthropogenic emissions of CFCs and halons compared to the Antarctic [9], which leads to a much less significant enhancement of the UV solar forcing reaching the Arctic surface; b) the dominant influence of oceanic iodine sources on the coastal Renland location, which determines the iodine accumulation. Indeed, due to the natural feedback mechanism between tropospheric ozone and inorganic iodine oceanic emissions [11], the enhanced ozone pollution background within the northern Atlantic ocean led to a threefold increase of iodide accumulation over the Arctic since 1950 [10]; c) higher snow accumulation (about 20 times larger than for Dome C) that allows the sudden burial of seasonal snow with lower exposure to UV and enhanced iodine record preservation. Thus, while the Renland ice core iodine record follows the changes in tropospheric ozone (which drives the enhancement in iodine emissions from the ocean in the Northern Hemisphere), the Dome C iodine record mirrors stratospheric ozone (which in turn drives the enhancement in UV radiation reaching the Antarctic surface), highlighting the wide variety of geochemical influences affecting iodine chemistry.

S5. CAM-Chem configuration and validation

The CAM-Chem VSL setup includes geographically distributed and seasonally–dependent natural oceanic emissions of five bromocarbons (VSL^{Br} = $CHBr_3$, CH_2Br_2, CH_2BrCl, $CHBrCl_2$, $CHBr_2Cl$) and four iodocarbons (VSL^{I} = CH_3I, CH_2ICl, CH_2IBr, CH_2I_2), whose oceanic flux is assumed to remain constant during the whole modelling period[12,13]. This includes the additional inorganic chlorine and bromine contribution arising from the so-called sea-salt dehalogenation recycling occurring in the marine boundary layer and the free troposphere [14]. Abiotic oceanic sources of inorganic iodine (HOI and I_2) arising from the ozone mediated oxidation of aqueous iodide are also considered and computed on-line based on modelled surface ozone abundance [11]. The ice-related polar tropospheric sources of organic [15] and inorganic [16] halogens are not considered here. Monthly varying zonally averaged distributions and lower boundary conditions of chlorinated CFCs and HCFCs (CH_3Cl, CH_3CCl_3, CCl_4, CFC–11, CFC–12, CFC–113, HCFC–22, CFC–114, CFC–115, HCFC–141b, HCFC–142b), as well as brominated longed-lived halons (CH_3Br, H–1301, H–1211, H–1202, and H–2402), were considered following the A1 halogenated ozone–depleting substances emissions scenario from WMO Ozone Assessment Report [17,18]. The surface concentrations of CO_2, CH_4, H_2 and N_2O were specified following previous works [19,20].

The CAM–Chem configuration used here extends from the surface to approximately 40 km (3.5 hPa in the upper stratosphere) with 26 vertical levels and includes a horizontal resolution of 1.9º latitude by 2.5º longitude. The atmospheric chemistry and dynamics is computed on-line in free-running mode for the 1950-2010 time period, coupled to prescribed sea surface temperatures and sea ice distributions [21]. To have a reasonable representation of the overall stratospheric circulation, the integrated momentum that would have been deposited above the model top is specified by an upper boundary condition. Even

though the model top is relatively low compared to other climate models such as WACCM [22], CAM-Chem shows good ability at reproducing large-scale changes in climate and chemical composition in the stratosphere when forced with the observed sea-surface temperatures and surface concentrations of long-lived trace gases and ozone-depleting substances [13,21]. Indeed, CAM-Chem was part of CCMVal-2[23] and CMIP5 [24] inter-comparison projects, presenting ozone evolution trends and return date estimations that lie in the middle of the multi-model range.

Figure S2 shows a typical model evaluation of the ozone hole evolution based on the inter-comparison of the October mean Total Ozone Column within the southern polar cap (poleward of 63°S; TOC^{SP}) with respect to the SBUV (Solar Backscatter Ultraviolet) observations between 1950 and 2010[17,18]. The SBUV ground, balloon-borne and satellite-based merged data set was constructed from ozone profiles measured by individual SBUV instruments and include individual calibration adjustments [25]. Equivalent smoothing has been applied to model output and observations, considering a Hamming filter with an 11-year averaging window. The CAM-Chem October TOC^{SP} shows an excellent agreement with the observed trend in the total ozone reduction above Antarctica[13].

The modelled TOC changes above the closest model grid points to Dome C (TOC^{DC}: 74.84ºS; 122.5ºE) and Law Dome (TOC^{LD}: 66.46ºS, 112.48ºE) are also shown in Figure S2. Due to the northern location of Law Dome, closer to the latitudinal boundary of the stratospheric polar vortex that determines the ozone hole edge, the total ozone decrease in recent years is smaller than the trend observed at Dome C. Indeed, the October mean modelled TOC^{LD} prevailing during the pre-ozone hole period decreased only ~7% by 1988 ($\Delta TOC^{LD} \approx -30$DU, from 390 DU for the 1950-1973 period to 360 DU between 1980-1988). Comparatively, the maximum modelled total ozone reduction at Dome C during the whole modelled period reached approximately −60% ($\Delta TOC^{DC} \approx -200$DU for 1998 AD), suggesting a different trend in the iodine photo-activation efficiency affecting the post-depositional processes between the two Antarctic ice-records as described above in section S4 and shown in Figure S1.

S6. Seasonal, latitudinal and UV-wavelength dependence of the iodine photo-activation mechanism

The stratospheric and tropospheric radiative transfer in CAM-Chem is computed online and considers both long-wave and short-wave parameterizations, including wavelength integrated gas-phase photolysis rate computations for short-wavelengths and a look-up-table approach for long-wavelengths [21]. The complete wavelength grid includes 100 bins from 121 nm to 750 nm, with a spectral resolution ranging from less than 1 nm in the UV to 50 nm in the visible edge. Within the 280-350 nm bandwith considered in this work, each wavelength bin center is located at 281, 285, 289, 293, 296.75, 300.5, 304, 307, 310, 313, 316, 320, 325, 330, 335, 340, 345, 348.75, 352.5 nm, with a mean bandwith of of 4 nm. The 300.5 nm bin, which has a mean bandwidth of 3.625 nm, has been used for adressing the trend in the surface Actinic Flux (AF^{300}) at Dome C (Figure 2 in the main text). A sensitivity analysis of the 1950-2010 trend of the Actinic Flux at different wavelentghs within the 280-330 nm band is shown in Figure

S5b. Note that the CAM-Chem photolysis module was shown to be one of the most accurate modules used in Chemistry Climate Models intercomparisons[21] .

To evaluate the validity of considering Eq. (1) in the Methods section to compute the enhancement in the iodine photo activation efficiency (i.e., J-iodine) driven by the stratospheric ozone depletion observed between 1950-2010, we perform a sensitivity analysis by changing the wavelength interval used to performed the J-iodine intergration. Note that Kim et al., (2016)[26] reported an enhanced I_2 emision from frozen iodide solutions (both under a pure oxygen or mixed air environments) by exposure to UV-B radiation below 400 nm, but it was not determined whether there is a wavelength threshold requirement to initiate the photo-oxidative production of I_2. Indeed, recent quantum mechanics computations suggest that the iodine activation on ice involves the formation of the IO_2H intermediate species, which has slightly different photactivation bands for the S_3 (328 nm) and S_4 (308 nm) singlet states [27]. Thus, we fixed the lower wavelength range for the J-iodine integration at 280 nm and moved the upper wavelength limit between 297 nm and 325 nm (Figure S5). The mean normalized J-iodine for both I^--air and I^--$O_{2(aq)}$ solutions during the ozone hole period (1975-2010) increased between approximately 1.25 to up to 6.5 times with respect to the values prevailing before 1975 (Figure S5c). As the relative actinic flux enhancement between the pre- and post-1975 periods is maximized at lower wavelengths (Figure S5b), the J-iodine increase is larger for the narrower wavelength bandwidths (Figure S5c), although the net ammount of UV radiation reaching the surface is considerably reduced. On the contrary, the normalized J-iodine is insensitive to shifting the upper wavelength limit between 325 nm and 350 nm as the I^--O_2 complex absorption drops to zero above 330 nm[26], while at the same time the 1975-2010 evolution of actinic flux for $\lambda > 330$ nm) is not affected by the formation of the ozone hole (see Figure S5a). Further laboratory and field work is required to determine the exact wavelength threshold for UV-driven iodine photoactivation on the snowpack.

Eventhough the seasonal cycle of the ozone hole formation and closure embrasses the austral springtime months (September, October and November), the stratospheric ozone reduction due to CFCs and Halons during recent years extends also into the early summer[28]. In addition, the higher summertime position of the Sun over the horizon (i.e., larger Solar Zenith Angle, SZA) with respect to spring, results in that even under a thicker ozone layer the AF intensity reaching the Antarctic during the summer months to be similar or even larger than during spring. Thus, although the largest relative enhancement of the J-iodine(October) is maximized during spring, the modeled December and January photoactivation efficiency also contributes to the overall annual trend. Figures S6a and S6b compares the trends in the intensity of $AF^{surf}(\lambda,yr)$ at different wavelengths as well as the corresponding changes on the computed J-iodine values for the different fractions of the sunlit period. Thus, and even though the Antarctic ozone hole is a springtime phenomenon, the complex interplay between ozone reduction during spring, increased radiative intensity during the summer, as well as the wavelength dependence of both iodide absorbance and AF radiative transference under a varying ozone layer, justifies the

assumption that the complete sunlit period (i.e., spring + summer) must be considered to evaluate the photoactivation efficiency and re-emission of snow-trapped iodine over the inner Antarctic plateau.

Finally, Figures S6c and S6d show an equivalent intercomparison for the normalized actinic flux and J-iodine (280-313 nm) computed at different latitudes from the south pole to the ozone hole edge (i.e., the zonal mean J-iodine at each latitude was computed). Here it can be clearly seen that the normalized J-iodine enhancement is also very sensitive to the latitudinal band considered due to the large changes in the total ozone column for the different locations (i.e., see TOC^{DC} and TOC^{LD} in Figure S2), presenting larger normalized J-iodine trends as we move from the Antarctic coast into the South Pole (i.e., further inside the ozone hole center). This highlights the potential use of considering trends in iodine ice records drilled within the inner Antarctic plateau as a proxy for determining the evolution of past stratospheric ozone variations at centennial to millennial time scales.

S7. Possible effect of ice core sample transportation and snow sample processing on iodine loss.

In addition to all the processes described in the main text, we further discuss here the possible effect of sample storage and transport on iodine concentration. It has been reported that firn samples, being characterized by a lower density, can lead to significant iodine losses during storage[29]. However, this was reported only for layers having a density close to 0.83 kg L^{-1} (i.e. close to the firn to ice transition). In this record, the density values in the first 3.5 meters (i.e. when the iodine decrease was detected) slightly increased from 0.32 to 0.38 kg L^{-1} and this cannot explain the observed two-fold decrease in iodine concentration. If density had driven the decrease in iodine concentration, we should have expected an iodine-decreasing trend along the entire record and not only in the uppermost meters. Through the comparison of three different records, we also investigated whether the storage time might have played a role in explaining the observed iodine concentration change in the 1970s. The firn core samples were analysed 32 months after collection, Sp2013 samples were analysed 13 months after collection and Sp2017 samples were analysed 14 months after collection. A flat iodine profile with the lowest concentrations was recorded in the Sp2013 samples, where the time between sampling and the analysis was the shortest compared to the other records. This suggests that the time elapsed between the sampling and the analysis does not seem to play a role in affecting the iodine concentration and that the differences among the records are due to spatial variability. Lastly, iodine preservation during transport and storage in low-density samples was demonstrated in snow and firn sampling studies that detected iodine day-to-night variability and seasonal variability, respectively[8,30]. A key parameter that can enhance and activate iodine volatilization from snow\firn\ice samples is the exposure to light[7] but all the samples from both the shallow core and the snow pits were stored under dark conditions until they were processed and analysed. We underline that correct sample storage is fundamental to minimize iodine losses from ice core and snow samples. To assure reliable sample representativeness, the cold chain (temperature equal or below -20°C) as well as dark conditions must be guaranteed for the entire transportation process.

Iodine can also be lost during the analysis from melted ice samples. To avoid this risk, the samples were stored at temperature below -20°C and melted only immediately before the analysis. Any possible iodine loss was evaluated through the analysis of selected samples multiple times during the analytical run (approximately 12 hours), without detecting any significant iodine loss.

SUPPLEMENTARY FIGURES

Figure S1. Iodine ice core records from Arctic and Antarctica. The available long-term ice core iodine records are rather limited. The only ice core that covers the same period covered by our Dome C record is the ReCAP ice core[10], collected from the eastern coast of Greenland (Arctic). From Antarctica only two records exist: Law Dome[8] and Talos Dome. The Talos Dome ice core cannot be used for a comparison since the most recent sample analyzed is dated 4 ky BP[7]. Similarly, the Law Dome record ends in 1988.

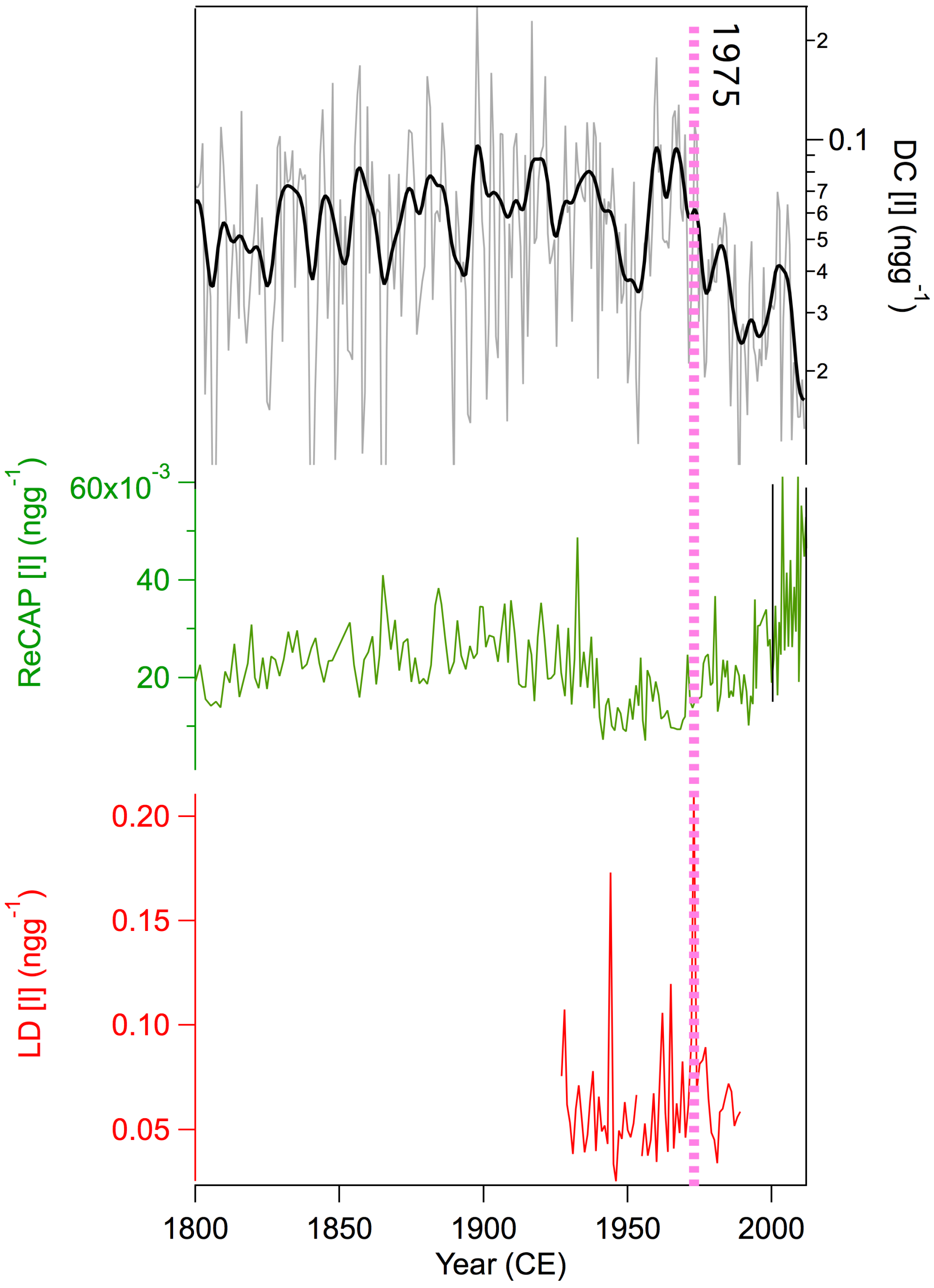

Figure S2._Validation of the CAM-Chem ozone hole evolution: Temporal evolution of the modelled total ozone column averaged within the southern polar cap (TOC^{SP}, poleward of 63ºS, red) in comparison to the merged satellite SBUV (Solar Backscatter Ultraviolet) database (black). Thin coloured lines indicate the October monthly mean for each year, while thick lines show a Hamming smoothing filter with an 11-year window. The October TOC^{SP} monthly average during each year is usually considered to validate the model performance in reproducing the mean ozone hole evolution since its appearance in the mid-1970's. The blue and green lines show the mean total ozone column at the model gridpoints closest to Dome C (TOC^{DC}) and Law Dome (TOC^{LD}), respectively, highlighting the different influences of the total ozone reduction between a station in the inner Antarctic plateau and other locations closer to the Antarctic coast.

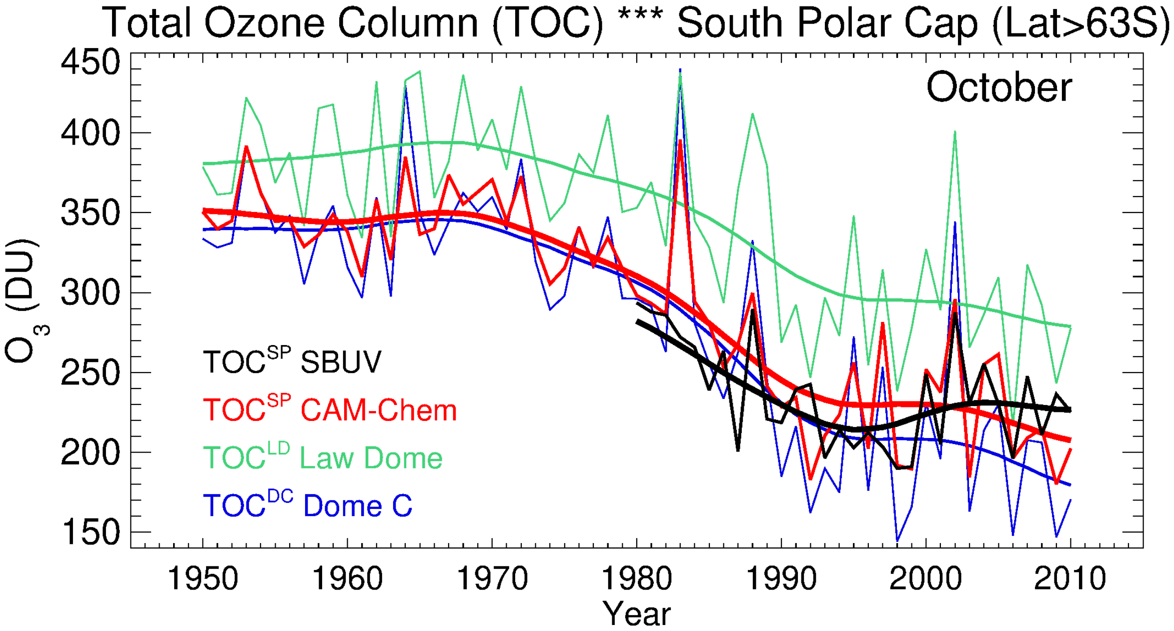

Figure S3. Snow physical proprieties in the Sp2017. Iodine concentration (ng g^{-1} - lower panel in green) compared with density (blue line – kg L^{-1}), hardness index (dark red - hand test and Swiss Rammesonde method) and grain size (in mm - upper panel black line).

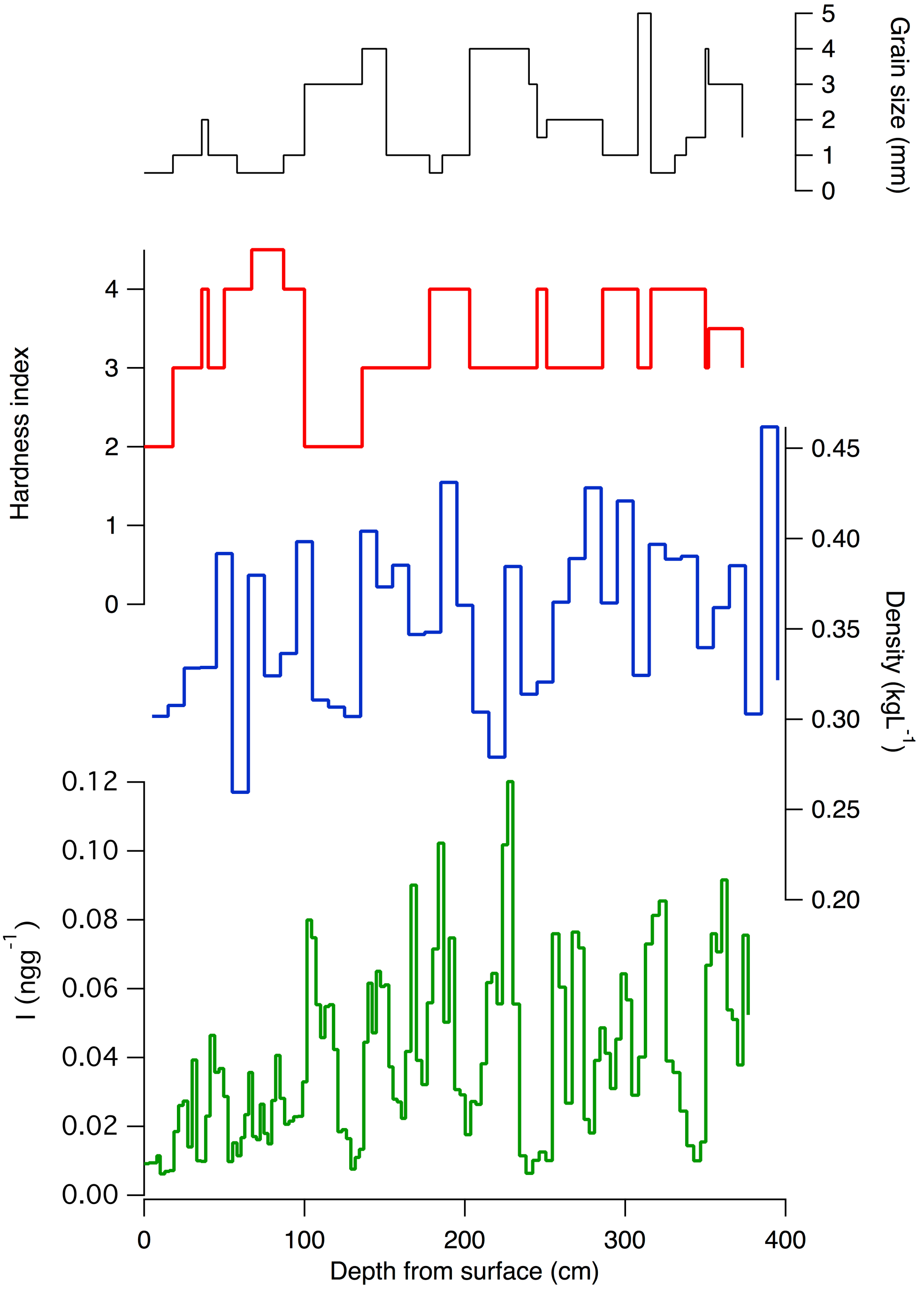

Figure S4. Photochemical species in the Dome C snow pack. The iodine concentration profile obtained from the ice core (Ic2012), the snow pit 2017 (Sp2017) and snow pit 2013 (Sp2013) (lower panel, in red, blue and black respectively) were compared with the NH_4^+, NO_3^-, MSA, Cl^- and acetate records obtained from the 2017 snow pit. All these species can experience photochemical post depositional processes. However, while iodine shows a decreasing trend, the other species, except acetate that does not show any trend, have an opposite behaviour with higher concentration in the upper meters of the snow pack.

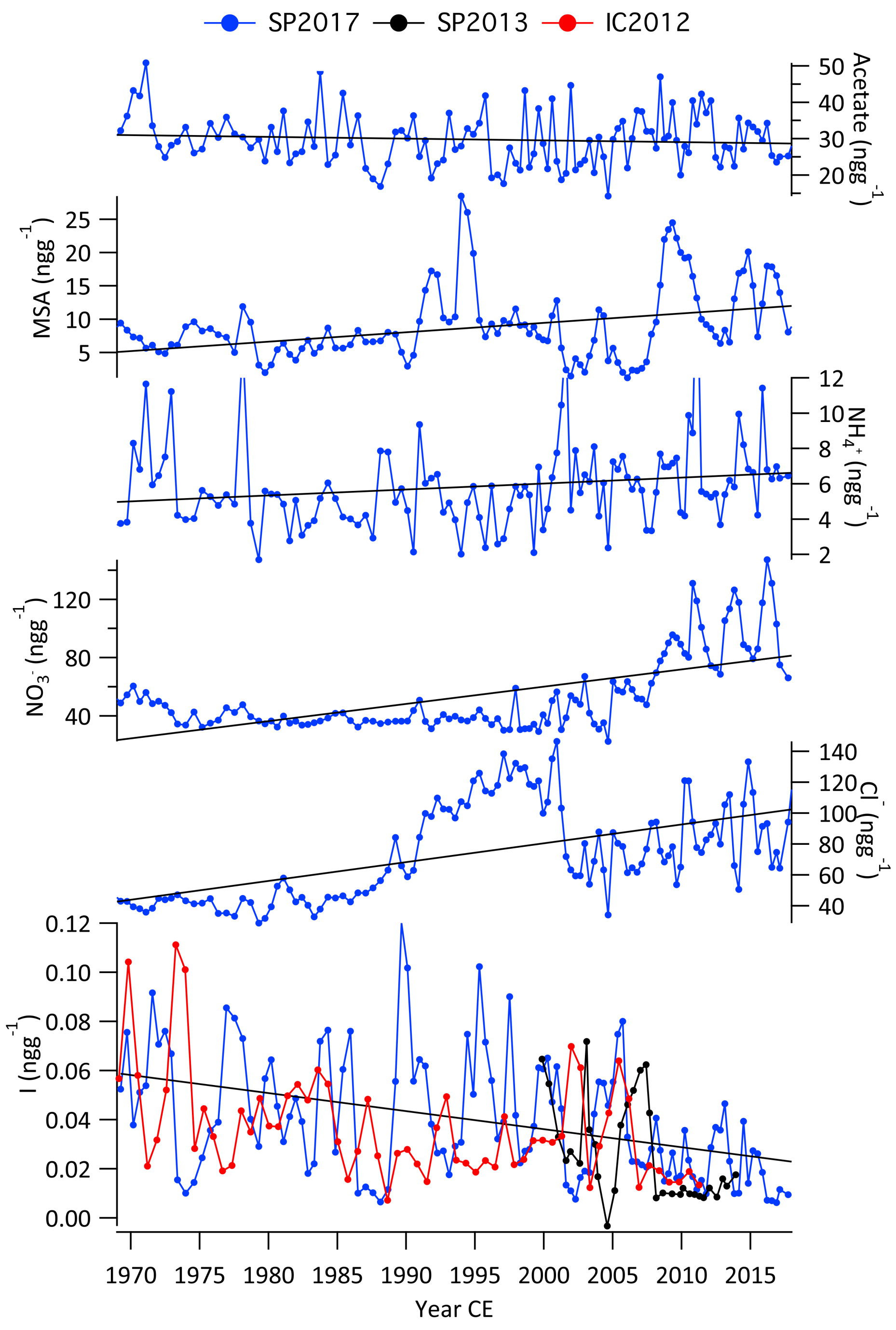

Figure S5. Wavelength sensitivity of iodine photo-activation efficiency (J-iodine): The J-iodine variation computed following Eq. 1 depends both on the wavelength interval considered as well as on the variation of the modelled Actinic Flux during each year: a) Measured absorbance spectra of a frozen iodide solution in equilibrium with oxygen ($I^{-}(O_2)$) and air (I-(air)) environment as a function of wavelength (right Y-axis), while the grey and black circles show the model actinic flux (AF) for each wavelength bin during 1960 and 2000 (left Y-axis), respectively. The light grey dashed vertical line indicates the lower limit of the bandwidth used for J-iodine integration (Eq. 1), whereas each coloured dashed vertical line indicates the change in the wavelength-bin used as upper limit; b) Annual variation of the sunlit mean AF between 1950 and 2010; c) Annual variation of the snow-trapped J-iodine relative to the mean J-iodine for the pre-1975 period.

The colour coding in panels b) and c) corresponds to the upper limit wavelength bin presented in panel a).

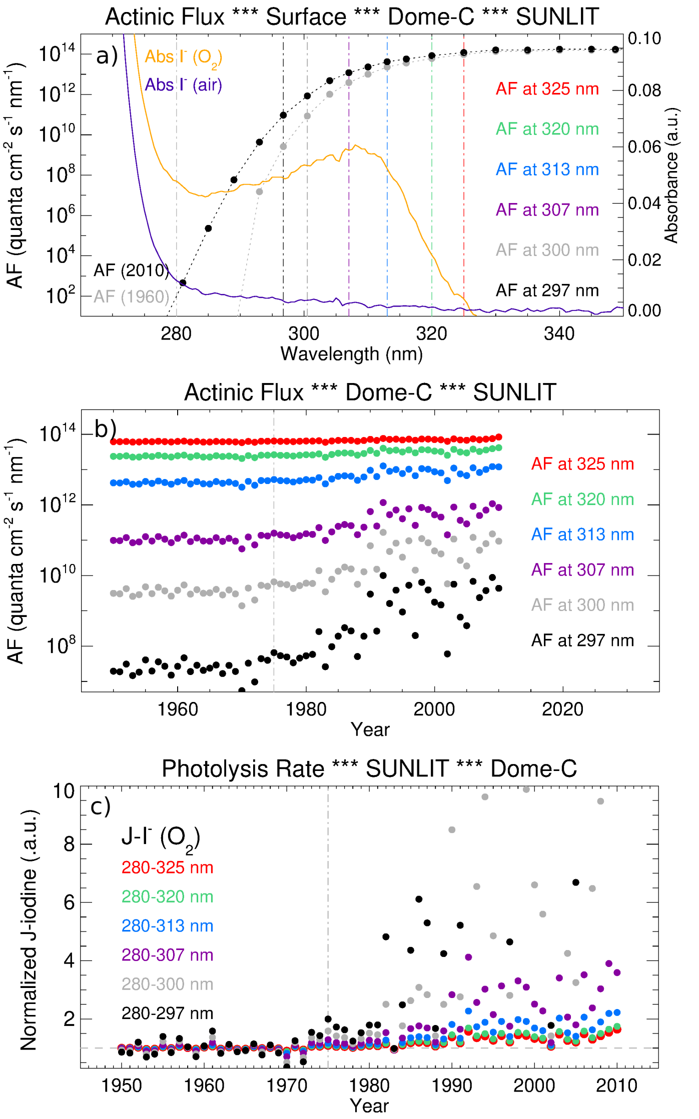

Figure S6. Seasonal and latitudinal dependence of iodine photo-activation efficiency (J-iodine): Annual evolution of modelled Actinic Flux at 300 nm (AF^{300}, left column) and normalized J-iodine efficiency within the 280-313 nm bandwidth (right column) between 1950 and 2010. Panels a) and b) (top row) highlight the change in the evolution of each magnitude for different months within the sunlit period. The normalized J-iodine efficiency maximizes during September and October where the largest changes in the Total Ozone Column (TOC) are observed, although during those springtime months the intensity of UV-radiation reaching the surface is smaller than during the summer. On contrast, during December and January the net AF^{300} maximizes due to the higher SZA, but the smaller change in TOC during the summertime months result in a smaller relative J-iodine enhancement. Panels c and d) (bottom row) show the latitudinal dependence the modelled AF^{300} and J-iodine (zonal means are considered for each latitude). The photo-activation efficiency during the post-1975 period decreases from the South Pole moving towards northern latitudes driven by the rapid changes in actinic flux inside and outside the ozone hole edge usually located northward of 70ºS.

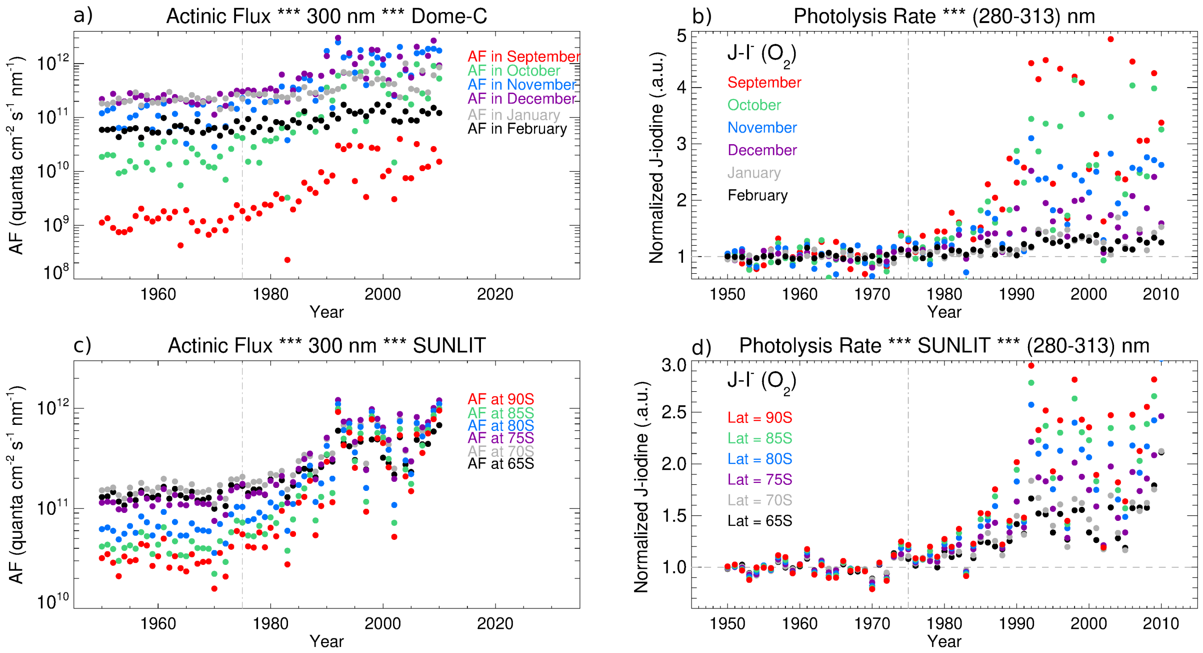

SUPPLEMENTARY TABLES

Table S1 – Comparison between the ice age scale calculated by Gautier et al. [1] and the ice scale calculated in this work.

	Gautier et al 2016			This work*		
	**Year**	Depth (m)	Depth (m w.e)	Depth (m)	Depth (m w.e.)	Age uncertainty(±)
**Surface**	2010	0	0	0.12	0.019	1 year
**Pinatubo**	1992	1.53	0.49	1.65	0.53	2 year
**Krakatua**	1884	8.75±0.09	3.29	9.01	3.37	4 years
**Cosigüina**	1835	11.71±0.16	4.68	11.82	4.59	5 years
**Tambora**	1816	12.67±0.12	5.09	12.82	5.07	5 years
**UE 1809**	1809	13.1±0.14	5.30	13.22	5.25	5 years

*based from the results obtained from Gautier et al. and the surface snow accumulation

Table S2. Comparison among the iodine concentrations (express a ng g^{-1}) retrieved from the ice core, the snow pit dug in 2017 (Sp2017) and the snow pit dug in 2013 (Sp2013). The periods shown in brackets indicate the estimate total time coverage of each record.

	Ice core (1800-2012)	SP2017 (1969 – 2018)	SP2013 (1999 – 2014)
1999-2012	0.032 ± 0.019	0.029 ± 0.018	0.026 ± 0.020
1969-2012	0.037 ± 0.022	0.039 ± 0.025	N\A
1800-1969	0.060 ± 0.038	N\A	N\A

Table S3. Snow temperature in the upper 10 m of Dome C snow pack during the year[31].

Month\depth	**0.05**	**0.10**	**0.25**	**0.50**	**0.75**	**1.00**	**1.50**	**2.00**	**2.50**	**3.00**	**4.00**	**5.00**	**6.00**	**8.00**	**10.00**
**1**	-29.77	-30.79	-23.24	-33.75	-36.32	-38.74	-46.80	-50.24	-51.85	-53.02	-54.30	-55.18	-55.01	-54.29	-54.65
**2**	-40.86	-40.72	-42.16	-38.95	-39.46	-40.01	-44.71	-47.57	-49.26	-50.78	-52.75	-54.28	-54.64	-54.23	-54.74
**3**	-53.48	-52.90	-53.75	-48.88	-48.20	-47.38	-47.73	-49.00	-49.64	-50.24	-51.90	-53.50	-54.21	-54.12	-54.79
**4**	-61.70	-61.31	-62.83	-57.71	-56.88	-55.59	-53.03	-52.66	-52.09	-51.20	-51.86	-53.01	-53.31	-53.86	-54.73
**5**	-61.93	-61.74	-63.18	-59.04	-58.73	-57.96	-55.79	-55.33	-54.32	-52.76	-52.62	-53.12	-53.34	-53.61	-54.60
**6**	-64.95	-64.67	-65.05	-61.54	-61.16	-60.29	-57.62	-56.88	-55.72	-53.97	-53.45	-53.56	-53.54	-53.48	-54.48
**7**	-66.44	-66.23	-66.12	-63.45	-63.15	-62.32	-59.11	-58.10	-56.67	-54.91	-54.12	-54.01	-53.84	-53.46	-54.39
**8**	-64.43	-64.35	-64.19	-62.15	-62.25	-61.88	-59.36	-58.73	-57.31	-55.71	-54.83	-54.52	-54.22	-53.53	-54.34
**9**	-62.53	-62.66	-61.36	-61.22	-61.61	-61.50	-59.32	-58.88	-57.52	-56.06	-55.18	-54.82	-54.47	-53.61	-54.33
**10**	-57.28	-57.64	-56.90	-57.29	-58.21	-58.70	-58.65	-58.84	-57.73	-56.39	-55.58	-55.16	-55.02	-53.73	-54.35
**11**	-45.35	-46.81	-39.97	-49.46	-51.18	-52.59	-56.79	-57.91	-57.48	-56.41	-55.90	-55.56	-55.11	-53.89	-54.37
**12**	-33.55	-35.09	-22.05	-39.42	-42.26	-44.78	-52.00	-54.64	-55.40	-55.42	-55.69	-55.78	-55.30	-54.16	-54.50
**min**	-66.44	-66.23	-66.12	-63.45	-63.15	-62.32	-59.36	-58.88	-57.73	-56.41	-55.90	-55.78	-55.30	-54.29	-54.79
**max**	-29.77	-30.79	-22.05	-33.75	-36.32	-38.74	-44.71	-47.57	-49.26	-50.24	-51.86	-53.01	-53.31	-53.46	-54.33
**deltaT**	36.67	35.44	44.06	29.69	26.83	23.59	14.65	11.31	8.47	6.17	4.03	2.77	1.99	0.83	0.46

REFERENCES

1 Gautier, E., Savarino, J., Erbland, J., Lanciki, A. & Possenti, P. Variability of sulfate signal in ice core records based on five replicate cores. *Clim. Past* **12**, 103-113, doi:10.5194/cp-12-103-2016 (2016).

2 Frezzotti, M. *et al.* Spatial and temporal variability of snow accumulation in East Antarctica from traverse data. *J Glaciol* **51**, 113-124, doi:10.3189/172756505781829502 (2005).

3 Spolaor, A. *et al.* Feedback mechanisms between snow and atmospheric mercury: Results and observations from field campaigns on the Antarctic plateau. *Chemosphere* **197**, 306-317, doi:https://doi.org/10.1016/j.chemosphere.2017.12.180 (2018).

4 Spolaor, A. *et al.* Sea ice dynamics influence halogen deposition to Svalbard. *The Cryosphere* **7**, 1645-1658, doi:10.5194/tc-7-1645-2013 (2013).

5 Barbaro, E. *et al.* Particle size distribution of inorganic and organic ions in coastal and inland Antarctic aerosol. *Environmental Science and Pollution Research* **24**, 2724-2733, doi:10.1007/s11356-016-8042-x (2017).

6 Taylor, W. A. Change-Point Analysis: A Powerful New Tool For Detecting Changes. (2000).

7 Spolaor, A. *et al.* Halogen species record Antarctic sea ice extent over glacial-interglacial periods. *Atmos. Chem. Phys.* **13**, 6623-6635, doi:10.5194/acp-13-6623-2013 (2013).

8 Vallelonga, P. *et al.* Sea-ice-related halogen enrichment at Law Dome, coastal East Antarctica. *Clim. Past* **13**, 171-184, doi:10.5194/cp-13-171-2017 (2017).

9 Chipperfield, M. P. *et al.* Detecting recovery of the stratospheric ozone layer. *Nature* **549**, 211-218, doi:10.1038/nature23681 (2017).

10 Cuevas, C. A. *et al.* Rapid increase in atmospheric iodine levels in the North Atlantic since the mid-20th century. *Nature Communications* **9**, 1452, doi:10.1038/s41467-018-03756-1 (2018).

11 Prados-Roman, C. *et al.* A negative feedback between anthropogenic ozone pollution and enhanced ocean emissions of iodine. *Atmos. Chem. Phys.* **15**, 2215-2224, doi:10.5194/acp-15-2215-2015 (2015).

12 Ordóñez, C. *et al.* Bromine and iodine chemistry in a global chemistry-climate model: description and evaluation of very short-lived oceanic sources. *Atmos. Chem. Phys.* **12**, 1423-1447, doi:10.5194/acp-12-1423-2012 (2012).

13 Fernandez, R. P., Kinnison, D. E., Lamarque, J. F., Tilmes, S. & Saiz-Lopez, A. Impact of biogenic very short-lived bromine on the Antarctic ozone hole during the 21st century. *Atmos. Chem. Phys.* **17**, 1673-1688, doi:10.5194/acp-17-1673-2017 (2017).

14 Fernandez, R. P., Salawitch, R. J., Kinnison, D. E., Lamarque, J. F. & Saiz-Lopez, A. Bromine partitioning in the tropical tropopause layer: implications for stratospheric injection. *Atmos. Chem. Phys.* **14**, 13391-13410, doi:10.5194/acp-14-13391-2014 (2014).

15 Abrahamsson, K., Granfors, A., Ahnoff, M., Cuevas, C. A. & Saiz-Lopez, A. Organic bromine compounds produced in sea ice in Antarctic winter. *Nature Communications* **9**, 5291, doi:10.1038/s41467-018-07062-8 (2018).

16 Fernandez, R. P. *et al.* Modeling the Sources and Chemistry of Polar Tropospheric Halogens (Cl, Br, and I) Using the CAM-Chem Global Chemistry-Climate Model. *Journal of Advances in Modeling Earth Systems* **11**, 2259-2289, doi:10.1029/2019MS001655 (2019).

17 WMO. Scientific Assessment of Ozone Depletion: 2010, Global Ozone Research and Monitoring Project-Report No. 52. (Geneva, Switzerland, 2011).

18 WMO. Scientific Assessment of Ozone Depletion: 2010, Global Ozone Research and Monitoring Project-Report No. 55. (Geneva, Switzerland, 2014).

19 Meinshausen, M. *et al.* The RCP greenhouse gas concentrations and their extensions from 1765 to 2300. *Climatic Change* **109**, 213, doi:10.1007/s10584-011-0156-z (2011).

20 Eyring, V., Lamarque, J.–F., Hess, P., Arfeuille, F., Bowman, K., Chipperfield, M. P., Duncan, B., Fiore, A., Gettelman, A., Giorgetta, M. A., Granier, C., Hegglin, M., Kinnison, D., Kunze, M., Langematz, U., Luo, B., Martin, R., Matthes, K., Newman, P. A., Peter, T., Robock, A., Ryerson, T., Saiz–Lopez, A., Salawitch, R., Schultz, M., Shepherd, T. G., Shindell, D., Stähelin, J., Tegtmeier, S., Thomason, L., Tilmes, S., Vernier, J.–P., Waugh, D. W. and Young, P. J. . Overview of IGAC/SPARC Chemistry–Climate Model Initiative (CCMI) Community Simulations in Support of Upcoming Ozone and Climate Assessments. 48–66 (2013).

21 Lamarque, J. F. *et al.* CAM-chem: description and evaluation of interactive atmospheric chemistry in the Community Earth System Model. *Geosci. Model Dev.* **5**, 369-411, doi:10.5194/gmd-5-369-2012 (2012).

22 Kinnison, D. E. *et al.* Sensitivity of chemical tracers to meteorological parameters in the MOZART-3 chemical transport model. *Journal of Geophysical Research: Atmospheres* **112**, doi:10.1029/2006JD007879 (2007).

23 Eyring, V. *et al.* Multi-model assessment of stratospheric ozone return dates and ozone recovery in CCMVal-2 models. *Atmos. Chem. Phys.* **10**, 9451-9472, doi:10.5194/acp-10-9451-2010 (2010).

24 Eyring, V. *et al.* Long-term ozone changes and associated climate impacts in CMIP5 simulations. *Journal of Geophysical Research: Atmospheres* **118**, 5029-5060, doi:10.1002/jgrd.50316 (2013).

25 Frith, S. M. *et al.* Recent changes in total column ozone based on the SBUV Version 8.6 Merged Ozone Data Set. *Journal of Geophysical Research: Atmospheres* **119**, 9735-9751, doi:10.1002/2014JD021889 (2014).

26 Kim, K. *et al.* Production of Molecular Iodine and Tri-iodide in the Frozen Solution of Iodide: Implication for Polar Atmosphere. *Environ Sci Technol* **50**, 1280-1287, doi:10.1021/acs.est.5b05148 (2016).

27 Baek, Y. S. *et al.* Entangled iodine and hydrogen peroxide formation in ice. *Phys Chem Chem Phys*, doi:10.1039/D0CP02966A (2020).

28 Solomon, S. Stratospheric ozone depletion: A review of concepts and history. *Rev Geophys* **37**, 275-316, doi:10.1029/1999RG900008 (1999).

29 Legrand, M. *et al.* Alpine ice evidence of a three-fold increase in atmospheric iodine deposition since 1950 in Europe due to increasing oceanic emissions. *Proceedings of the National Academy of Sciences* **115**, 12136, doi:10.1073/pnas.1809867115 (2018).

30 Spolaor, A. *et al.* Diurnal cycle of iodine, bromine, and mercury concentrations in Svalbard surface snow. *Atmos. Chem. Phys.* **19**, 13325-13339, doi:10.5194/acp-19-13325-2019 (2019).

31 Macelloni, G. *et al.* Ground-Based L-Band Emission Measurements at Dome-C Antarctica: The DOMEX-2 Experiment. *IEEE Transactions on Geoscience and Remote Sensing* **51**, 4718-4730, doi:10.1109/TGRS.2013.2277921 (2013).
